# Using Highlighting to Train Attentional Expertise

**DOI:** 10.1371/journal.pone.0146266

**Published:** 2016-01-08

**Authors:** Brett Roads, Michael C. Mozer, Thomas A. Busey

**Affiliations:** 1 Department of Computer Science and Institute of Cognitive Science, University of Colorado Boulder, Boulder, Colorado, United States of America; 2 Department of Psychological and Brain Sciences, Indiana University, Bloomington, Indiana, United States of America; VU University Amsterdam, NETHERLANDS

## Abstract

Acquiring expertise in complex visual tasks is time consuming. To facilitate the efficient training of novices on where to look in these tasks, we propose an attentional highlighting paradigm. Highlighting involves dynamically modulating the saliency of a visual image to guide attention along the fixation path of a domain expert who had previously viewed the same image. In Experiment 1, we trained naive subjects via attentional highlighting on a fingerprint-matching task. Before and after training, we asked subjects to freely inspect images containing pairs of prints and determine whether the prints matched. Fixation sequences were automatically scored for the degree of expertise exhibited using a Bayesian discriminative model of novice and expert gaze behavior. Highlighted training causes gaze behavior to become more expert-like not only on the trained images but also on transfer images, indicating generalization of learning. In Experiment 2, to control for the possibility that the increase in expertise is due to mere exposure, we trained subjects via highlighting of fixation sequences from novices, not experts, and observed no transition toward expertise. In Experiment 3, to determine the specificity of the training effect, we trained subjects with expert fixation sequences from images other than the one being viewed, which preserves coarse-scale statistics of expert gaze but provides no information about fine-grain features. Observing at least a partial transition toward expertise, we obtain only weak evidence that the highlighting procedure facilitates the learning of critical local features. We discuss possible improvements to the highlighting procedure.

## Introduction

Individuals spend a majority of their waking hours performing complex visual tasks and many occupations specifically require operating in challenging visual environments, e.g., monitoring multiple stock-exchange status displays, controlling air traffic, screening baggage, examining fingerprints, inspecting medical images, driving trucks, and performing surgery. Yet, acquiring visual expertise in any task domain is challenging and time-consuming.

Visual expertise might be decomposed into two interacting abilities: *attentional expertise*—knowing where to attend in complex, cluttered scenes—and *procedural expertise*—knowing what to do with the information gathered at the focus of attention. Acquiring these two abilities poses a circular challenge: individuals cannot learn what features and locations in the environment are task-relevant until they understand how the information should be integrated and processed, but individuals cannot learn how to process information until they identify relevant feature locations.

Studying the acquisition of visual expertise is quite challenging due to the interaction between the attentional and procedural skills required. Can one type of skill be studied in isolation? It does not make sense to study procedural expertise in the absence of attentional expertise, because attentional expertise provides the visual representations on which classification and judgment procedures operate. However, the reverse is not true: in principle, attentional expertise can be acquired in the absence of procedural expertise, as in, for example, a situation where one is instructed to classify based on color but not told the classification rule. Once novices have learned where and to what to attend, expert-like attention logically supports the acquisition of procedures an expert must perform.

Indeed, past work provides encouraging indications that guiding attention can guide higher-order cognitive processing [1,2,3,4,5,6,7,8]. For example, Grant and Spivey [2] noted that certain fixation patterns predict success on the tumor-and-lasers radiation problem, and simply cueing the critical locations increased the probability of success. Although subjects had no top-down guidance or goals that steered attention to a critical location, the mere act of attending to the location was sufficient to increase the likelihood of the relevant insight.

Our long-term goal is to develop procedures that improve training of visual expertise. Just as visual expertise might be decomposed into attentional and procedural skills, training of visual expertise might be decomposed similarly. Given the dependencies between attentional and procedural expertise we just discussed, it seems like a sensible first step to focus on the challenge of training attentional expertise. Thus, we wish to develop an efficient and relatively effortless means by which novices learn to deploy spatiotemporal attention in a task-appropriate manner. We explore a training paradigm that leverages expert knowledge in a perceptual learning paradigm that involves the following steps: (1) recording gaze dynamics of experts as they perform a particular task; (2) building a model that predicts locations experts are likely to inspect in specific images and task contexts; and (3) placing a novice in the visual environment and having them perform the task while highlighting predicted locations of interest via saliency manipulations.

This paradigm, which we refer to as *attentional highlighting*, addresses two challenges that arise when using experts to assist in the training of novices. First, expert knowledge is often procedural and implicit, and experts are limited in their ability to articulate their strategies [9,10]. For example, when experts fail to report abnormalities in medical images, fixation statistics still discriminate between missed abnormalities and abnormality-free areas (see [11] for a review). This finding suggests that expert gaze behavior reflects additional implicit knowledge that is not readily verbalizable. Attentional highlighting avoids the issue of knowledge accessibility by analyzing where experts are looking instead of asking experts to verbally report their strategies; expert fixations indicate the locations and features that are important for accomplishing the task. Second, verbal instruction may be unhelpful or even harmful to a trainee because it interferes with the deployment of attention and the natural pace of perceptuomotor behavior while performing a task. With attentional highlighting, verbal instructions are replaced by saliency enhancements, which leverage individuals’ rapid and automatic machinery for directing gaze.

This paper explores whether the acquisition of attentional expertise can be facilitated via highlighting—guiding attention by dynamically modulating saliency during training. Because individuals are capable of learning statistical correlations in visual information for statistically structured sequences of objects [12,13], task-irrelevant perceptual information [14], and visuospatial context [15], attending to expert fixation locations may be sufficient to train novices to deploy attention in a task-relevant manner.

Highlighting has been used to boost performance in problem solving and memory tasks. For example, in a collaborative puzzle-solving task involving a novice and an expert, Velichkovsky [7] observed improved performance when either the novice is cued to the expert partner’s fixation sequence, or vice versa. However, highlighting in conjunction with a verbal description of a solution procedure can impair a novice’s performance [16]. In the domain of recognition memory, yoking fixations at encoding and test improves performance, although replaying other-observer fixations at test is as effective as replaying same-observer fixations, and scanpath order does not matter [17]. In perception, Litchfield et al. [18] showed that novice radiologists benefit from viewing another individual’s scanpath.

Recently, attentional guidance has been used as a training method. Nalanagula, Greenstein, and Gramopadhye [19] trained novices to detect defects in circuit boards. Training included viewing three displays in which dynamic highlighting of location sequences was provided. Instead of using actual expert fixation sequences, the sequences used were based on the expert’s verbal expression of their search strategy given a trace of the raw saccade data. Dynamic highlighting of sequences during training led to better performance on the detection task over a control condition in which subjects viewed images without gaze cues. Unfortunately, the two conditions were not strictly matched for viewing time or controlled to ensure equal attention to the training images. Vine et al. [8] also found that guiding attention can expedite the learning of laparoscopic skills needed by surgeons. The task involved remotely manipulating balls into cups. Highlighting occurred via a mask overlaid on the field of view that occluded most of the scene. Under manual control, the experiment serially unmasked single locations as the objects at those locations became task-relevant. Because highlighted locations corresponded to task subgoals, highlighting served to sequence actions of the student in a task involving extended action sequences. Causer et al. [20] found that reviewing gaze behavior with subjects improved performance outcomes. As part of the training, subjects viewed videos of their gaze pattern and those of the top nationally ranked shotgun shooter. During the video sessions, researchers highlighted similarities and differences between the subject’s gaze behavior and the expert model. The eye gaze behavior of the training group become significantly more expert-like and shooting accuracy improved. Tomlinson, Howe, and Love [6] studied a video game in which players could select one of eight different status information formats for an on-screen display. Using a model of expert selection as a training companion, novices provided with contextually relevant information converged more rapidly on expert-like behavior.

Although these experiments establish that cueing novices to locations can speed training, a causal link between expert eye movements and performance is still uncertain. Are the eye movements exhibited by experts functional, i.e., do they contribute to the expert’s performance, or are they merely a byproduct of cognitive operations? To the extent that high-resolution vision is needed for performing a complex information processing task, clearly critical visual information must be foveated. For example, when radiologists are presented with x-ray images for a brief duration that prevented saccades, increasing the distance from the fixation point in the image to the tumor results in a monotonic drop in detection accuracy [21]. Although all individuals can attend to a location other than the locus of fixation [22], moving the eyes is more efficient than covertly shifting attention when performing complex visual tasks [23,24]. Beyond these arguments that fixation is efficient or necessary to obtain expert-level performance, a study by Thomas and Lleras [5] provides a causal link between fixation and performance. While subjects tried to solve the tumors and lasers problem, they were cued in a particular fixation sequence using an irrelevant detection task that was superimposed on the standard tumor diagram. Subjects were unaware that they were being cued in particular manner, but when cued in a solution-specific sequence—multiple saccades *across* different parts of the tumor boundary—subjects were more successful in solving the problem.

Based on the above results, we find it difficult to conceive that the fixations of experts are not a key contributing factor to their performance. Nonetheless, expertise has benefits that go beyond where the expert is fixating. When stimulus presentations are sufficiently brief that experts do not have time to make saccades, expert performance is often above chance, indicating that experts can utilize parafoveal and peripheral vision to attend to local features in a larger spatial context. Expert use of parafoveal and peripheral vision is directly implicated in domains such as chess, where experts do not always foveate on individual chess pieces, but instead foveate on empty squares that lie at the centroid of arrangement of pieces [25].

As a domain, we focus on the forensic task of comparing a pair of fingerprints. We begin by describing the fingerprint-matching domain, characterizing the fixation sequences of novices and experts, and constructing a model that reliably discriminates saccade sequences of novices and experts. With this model, we can conduct experiments and evaluate the degree to which training via attentional highlighting is effective in transitioning novices toward expert-like attentional control.

## Characterization of Fingerprint Examiners

**Fig 1:**
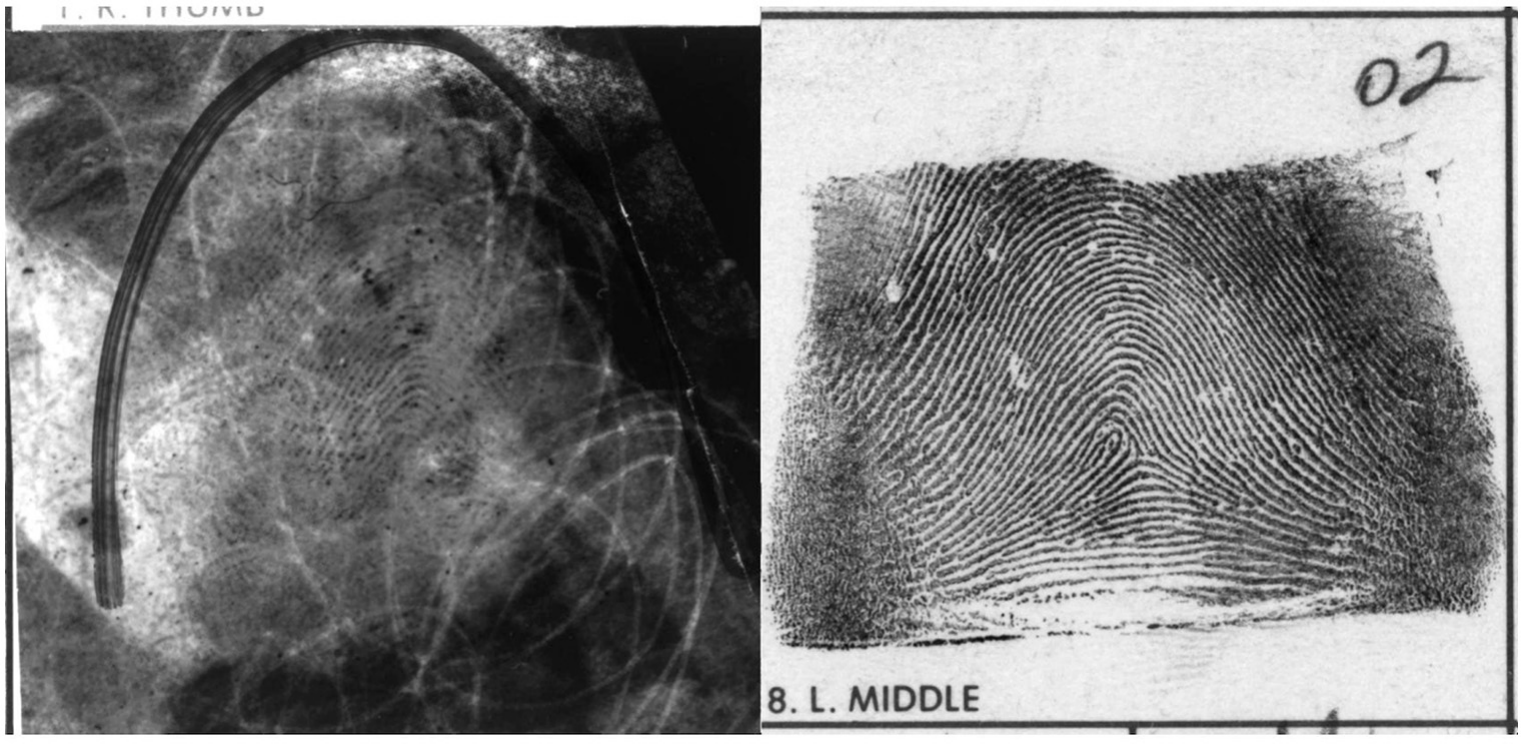
An example of realistic fingerprint casework taken from the National Institutes of Standards and Technology Special Database 27. This example demonstrates the noisy and partial nature of a latent print (left) compared to the matched inked print (right).

Fingerprint analysis is part of the broader field of forensic expertise, which involves the examination of partial or distorted trace evidence left at a crime scene. Fig 1 illustrates a typical fingerprint pair used in casework and adopts the common practice of placing the *latent print* on the left and the *inked print* on the right. Latent prints are obtained from crime scenes and are often distorted, partial, and overlaid on surplus visual information. However, inked prints are made under carefully controlled conditions to get as clean and consistent an impression as possible. Automated classification techniques have been developed to reliably match pairs of inked prints, but due to the variability and degradation of latent prints, matches involving a latent print and an inked print require expert human judgments.

Fingerprint examination expertise is acquired via a time-consuming training period; it can take 1–2 years until a trainee is allowed to carry out unsupervised casework [26]. However, following this training period, fingerprint examiners are exceedingly accurate compared to novices [27,28]. Due to the substantial training required, even modest gains in training efficiency would be beneficial. Forensic science is well-poised to benefit from novel training techniques that exploit research on perceptual expertise and cognitive science in general [29,30,31].

Although the expert examiner’s task will always involve latent prints, in the present work we focus on the task of comparing pairs of inked prints, for two reasons. First, initial training on noise-free and complete examples can benefit the learner [32]. Second, expert scanpaths are more consistent than novice scanpaths on inked prints [33]. In contrast, expert scanpaths are actually less consistent than novices on latent prints, possibly because the partial and noisy nature of latent prints elicits more idiosyncratic strategies [33]. Consequently, it is easier to evaluate the expertise of an examiner using pairs of inked prints.

**Fig 2:**
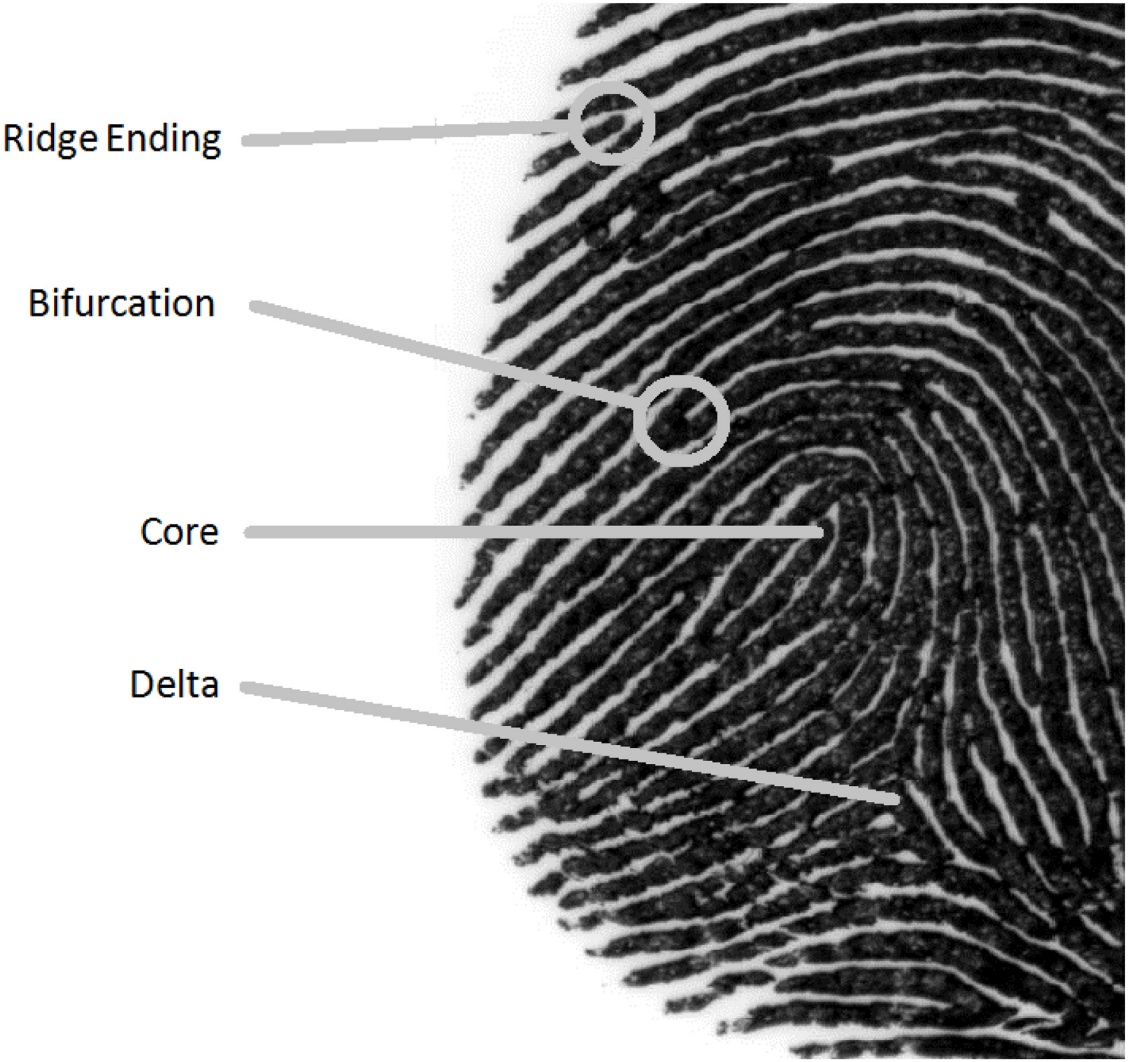
Close up of an inked print from the dataset used by Busey et al. [35]. Shown are the locations of the core and delta used in level one analysis and examples of the two fundamental minutiae—ridge endings and bifurcations—that are used in level-two analysis.

There is broad consensus among fingerprint experts that a fingerprint examination occurs at multiple levels of analysis [34]. At a coarse level of analysis, experts examine the overall ridge flow of the fingerprint. Two diagnostic ridge flow patterns are the core and the delta (Fig 2). While it is possible to reject a match at this coarse level, further analysis is necessary to confirm a match. At a fine level of analysis, experts zoom in to local discriminative features called *minutiae*; the two fundamental minutiae are ridge endings and bifurcations (Fig 2). Experts report that they typically rely on the intermediate level of analysis for making a judgment between two fingerprints [34].

### Eye Movement Data from Novice and Expert Fingerprint Examiners

To explicate differences between expert and novice fingerprint analysts we reanalyzed a dataset collected by Busey et al. [35]. The dataset consists of 26 matching inked fingerprint pairs and two mismatching pairs. (Matching pairs are a preferred and more informative source of data because ‘same’ judgments are typically slower and more deliberate than ‘different’ judgments.) Busey et al. [35] collected fixation data from 12 experts and 12 novices performing the matching task. In this study, experts were recruited at forensic identification conferences and laboratories, and novices were recruited from the Bloomington, Indiana community. Experts in the dataset had a reported average of 7.6 years of unsupervised latent print work (minimum 3 years, maximum 13 years). On each trial, subjects viewed a pair of fingerprints side by side, which we’ll refer to as the left and the right prints. Each trial was divided into three parts: for 5 s, the left print was presented alone, then for 5 s, the right print was presented alone, and finally for 10 s, both prints were presented simultaneously. After each trial, subjects were asked to indicate whether they believed the two prints came from the same source, different sources, or ‘unable to tell’.

**Fig 3:**
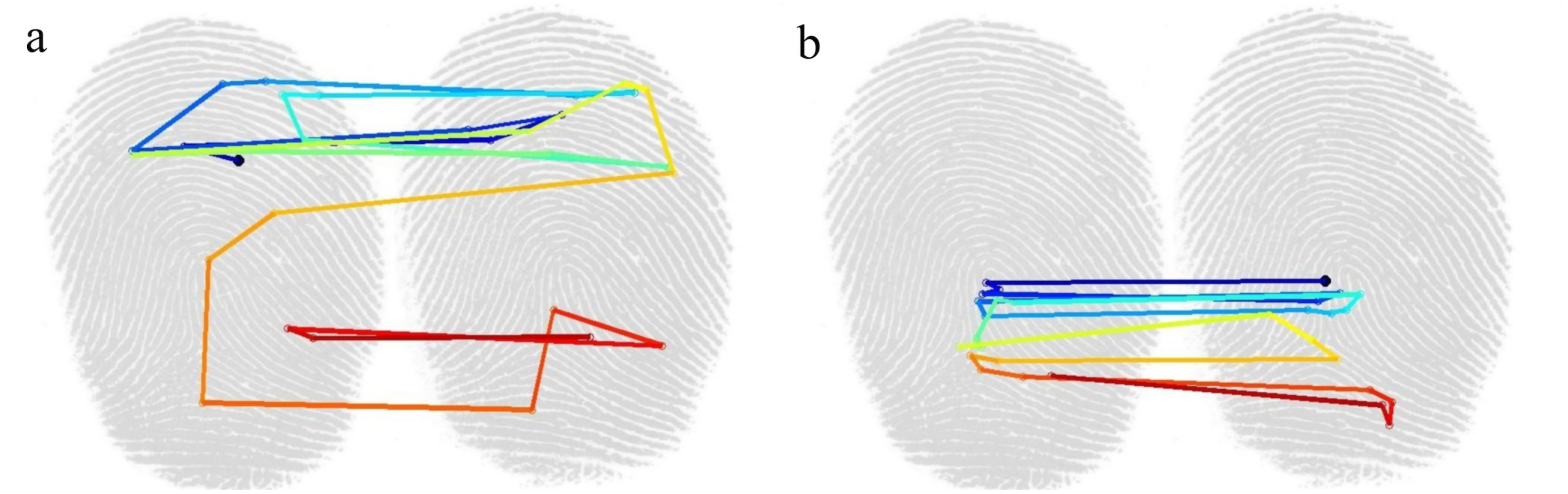
A prototypical scanpath of a novice (a) and an expert (b). The scanpath begins at the black dot on the end of the dark blue segment. The first fixation is located at the black dot on the right and the color scheme shows the expert's fixation sequence as it progresses through time, going from dark blue to yellow to dark red. This expert demonstrates a typical scanpath that concentrates on the core and delta regions of the fingerprint, diagnostic of level one analysis. In contrast, the novice exhibits less directed behavior.

Summary statistics of the dataset reveal no gross differences between expert and novice fingerprint examiners [35]. The total number of fixations (19.0 for experts vs. 18.1 for novices) and their mean durations (176 ms for experts, 174 ms for novices) are similar. Some differences can be found: experts have smaller saccade amplitudes on both the left and right print and experts make more saccades within prints than across prints than novices. The fact that experts make more saccades within prints is consistent with the idea that experts are more efficient at encoding and searching for new information, similar to the behavior exhibited by expert chess players [25] and expert medical diagnosticians [11]. Experts also have slightly more fixations on the left print than the right, which is consistent with the standard procedure for latent print examinations. In contrast to summary statistics, saccade targets and sequences indicate stark differences between novices and experts, as illustrated by Fig 3. We therefore focus on using stimulus-specific fixation patterns to discriminate novice and expert viewing behavior.

### Discriminative Modeling of the Degree of Expertise

In the experiments we describe in this article, subjects are shown fingerprint images and are instructed to inspect the images in order to determine whether or not the pair of fingerprints matches. The dependent measure in the experiments is the degree of attentional expertise exhibited by a subject before and after training. We use a subject’s fixation sequence to compute an attentional expertise score. The S1 Appendix presents the probabilistic model underlying this score.

Briefly, the model assigns a likelihood to a fixation sequence ***F*** given image ***I*** for a population *s* (novices or experts), denoted *p*(***F*** | *s*,  ***I***). If novices and experts systematically differ, a model of experts will assign a higher likelihood to expert sequences than to novice sequences, and a model of novices will do the opposite. By contrasting the predictions of the models, one can thus discriminate novice and expert attentional behavior. Specifically, Bayes’ rule can be used to classify a fixation sequence as having been produced by a novice or by an expert:

$$p\left(s|F,I\right)=\frac{p\left(F|s,I\right)p\left(s\right)}{\sum_{s^{\prime }}p\left(F|s\prime ,I\right)p\left(s^{\prime }\right)}$$ (1)

We use a measure related to Eq 1, *the log likelihood ratio (LLR)*, to characterize the degree of attentional expertise reflected in a subject’s fixation sequence.

The novice and expert likelihood models used in Eq 1, *p*(***F*** | *s* = novice,  ***I***) and *p*(***F*** | *s* = expert,  ***I***), describe the generative process underlying fixation sequences. However, the models should not be thought of as making theoretical claims about underlying cognitive processes. Instead, the models are simply being used as a black-box tool for discriminating novice and expert fixation sequences. In the S1 Appendix, we describe a family of models and, via previously collected data [35], identify the specific model that achieves the best inter-group discrimination.

Assuming models exist that can be used to assess the attentional expertise exhibited by subjects, we now address the main goal of our work: to develop and evaluate training procedures that yield a rapid and relatively effortless shift toward expert-like behavior by highlighting locations where an expert is likely to attend. We describe three experiments that explore attentional highlighting to train novice fingerprint examiners.

## Experiment 1: Expert Highlighting

**Fig 4:**
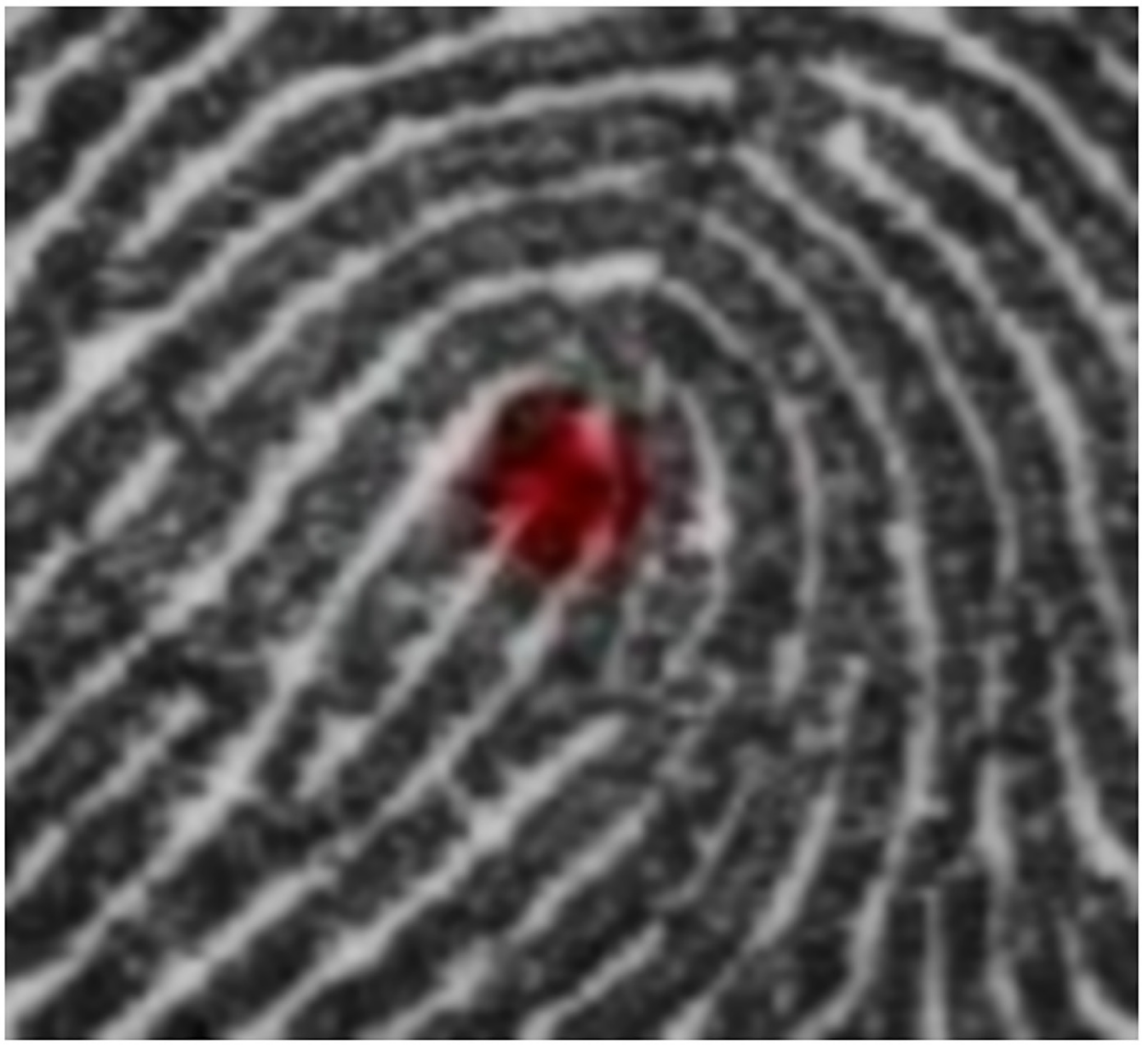
An example of the attentional highlighting used in Experiments 1–3. Each fixation location in a selected sequence shows attentional highlighting using a flashing red Gaussian intensity bump.

In Experiment 1, naive subjects were trained on fingerprint pairs to replicate the fixation sequence of one randomly selected expert from Busey et al.’s [35] dataset. (We chose to train each subject on a single expert to allow for the possibility of expert-specific idiosyncrasies [36]). Each training trial began with presentation of a fingerprint pair, followed by a cue to the expert’s first fixation location within the image. The cue consisted of a blinking red spot in the image background (Fig 4). When the subject made a saccade to the cue, as registered by an eye tracker, the cue shifted to the expert’s next fixation, and so forth. From the subject’s perspective, the training task involved following a sequence of red spots, sometimes jumping within a fingerprint and sometimes jumping to the other fingerprint. The training procedure preserves the order of fixations because there is potential information in the sequence itself [37], which may support learning. Experiments 2 and 3 involved a similar training procedure, but in Experiment 2, subjects were trained to follow the fixation sequence of a naive viewer, and in Experiment 3, subjects were trained to follow the fixation sequence that an expert made to a different stimulus rather than the current one. The rationale for these experiments will be explained shortly.

All three experiments included pre-training and post-training phases during which subjects performed free viewing of images. A comparison of fixation patterns pre- and post-training was used to assess the influence of training. During free viewing, the subjects’ task was to perform as a fingerprint analyst and examine the pair of prints in order to determine similarities and differences between them.

### Participants

Twelve subjects with normal or corrected to normal vision drawn from a paid subject pool at the University of Colorado, Boulder. Subjects were given $10 in compensation for their time. One subject was dropped because the eye tracker failed to reliably detect gaze shifts. The dropped subject was rerun.

### Apparatus and Materials

The experiment utilized a Tobii T60 XL eye tracker (24-inch 1920 x 1200 pixels widescreen monitor), an open source Talk2Tobii extension [38], the Psychophysics Toolbox extension [39], and MATLAB all running on an iMac computer. The 28 stimuli (each 1680x1050 pixels) consisted of two fingerprints side by side in 256 bit gray scale. (Two stimuli were discarded because some training data were corrupted on those two stimuli.) Stimuli were displayed pixel perfect on the eye tracker screen and subtended approximately 39.2 by 25.1 degrees of visual angle. The remainder of the screen surrounding the stimulus was displayed in black. The two fingerprints in each stimulus cannot be distinguished easily. Among the 28 possible stimuli, only two stimuli contained mismatching fingerprint pairs and not every subject was assigned a mismatching case.

### Procedure

The protocol for Experiments 1–3 was approved by the Institutional Review Board of the University of Colorado Boulder (Protocol 12–0661, "Visual Task Training Using Attentional Highlighting"). Written informed consent was obtained from all participants.

Prior to the beginning of the experiment, subjects were calibrated to maximize performance of the eye tracker. Subjects were seated so that their eyes were approximately 25 inches (63.5 cm) from eye tracker screen, as recommended by Tobii documentation, and the eye tracker’s height was adjusted so that the subject’s eyes were approximately level with the center of the eye tracker screen. Prior to any experiment-specific instructions, all subjects were verbally informed that blinking during the experiment was acceptable and that slight movement was permitted. The eye tracker was calibrated for each subject using a script that was slightly modified from a publicly available script that accompanied the Talk2Tobii software. Halfway through the experiment the eye tracker was calibrated again to account for drift.

At the initiation of the experiment, an automated program randomly assigned each subject 16 stimuli from 28 possible stimuli in the training dataset. These 16 stimuli made up the subject’s *stimulus set*. Eight of the 16 stimuli in the stimulus set were randomly assigned to the *training set* and the remaining eight were assigned to the *transfer set*. In addition to being randomly assigned stimuli, each of the 12 subjects was randomly paired with one of the 12 experts in the training dataset, in one-to-one correspondence. All attentional highlighting provided to a subject during the training phase utilized fixations from their matched expert and a given stimulus always showed the same fixation sequence.

**Fig 5:**
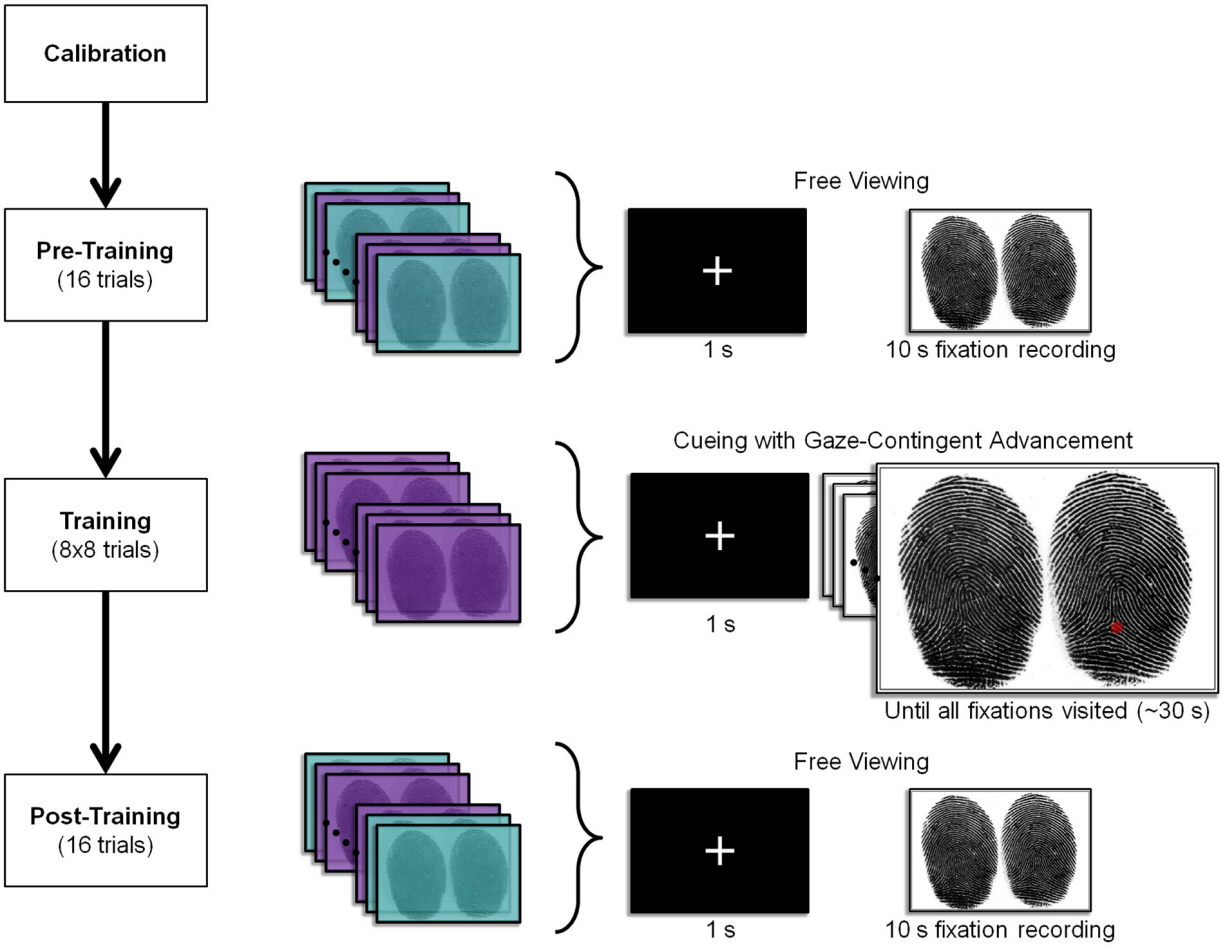
The four phases of Experiments 1–3. During the pre-test and post-test phases, subjects view images for 10 s and their fixations are recorded as they examine the prints. During the training phase, attentional highlighting guides fixations to locations of interest (red dot). Once subjects saccade to the location of the cue, the next fixation in the sequence is cued. Each trial begins with a fixation cue for 1 s preceding the onset of the fingerprint image. The fingerprint pairs shaded in purple represent the trained stimulus set, while the cyan fingerprint pairs represent the transfer stimulus set.

Following calibration, subjects received on-screen instructions which asked them to imagine that it was their first day on the job as a fingerprint analyst and they should look for similarities and differences between pairs of fingerprints displayed on the screen. After subjects indicated they were ready to continue, they were immediately presented with the on-screen instructions for the pre-training phase, informing them that they would begin by examining fingerprints without assistance. (The overall trial sequence is shown in Fig 5.)

The pre-training phase consisted of 16 trials, each involving free viewing of a stimulus for 10 s. On each trial, one of the 16 stimuli in the subject’s stimulus set was displayed, in randomized order. During the 10 s viewing period, the subject’s gaze was recorded. Prior to each trial, a white fixation cross was presented on a black background for 1 s. This pre-training phase was designed to mimic the simultaneous exposure period in Busey et al. [35] where two inked fingerprints were shown side by side. In the pre-training phase and the remainder of the experiment, subject responses were not collected because the focus of the study is on eye gaze behavior rather than on decision-making. Even without overt judgments, we were confident that the prints were carefully examined by virtue of the number of saccades produced on each trial, and the observation that the vast majority of saccades landed on one of the two prints.

Following the pre-training phase, subjects were trained using attentional highlighting over 64 trials, divided into eight blocks each consisting of the eight stimuli in the training set, randomized within a block. After blocks 2, 4, and 6, subjects were informed of their progress, reminded of their task and given the opportunity to take a quick break. Prior to the training phase, subjects were presented with on-screen instructions explaining that they would be examining pairs of fingerprints aided by attentional highlighting. They were told that they should look at the locations of a series of cues and that the trial would end when all of the cues in the sequence had been visited. They were not given any information about the source of the cues (i.e., that the locations were provided by an expert).

Each trial began with presentation of a white fixation cross on a black background. After 1 s, the cross was replaced by a pair of fingerprints centered on the cross location. Gaze-contingent attentional highlighting sequentially cued subjects to the locations of their assigned fixation sequence. The particular fixation sequence that each subject saw was determined by the expert they were assigned at the beginning of the experiment. For a given subject, all training trials used fixation sequences from the same expert viewing both images simultaneously. The onset of the first highlight (i.e., the first fixation in the expert sequence) coincided with the stimulus onset. Once the subject made a correct saccade to the cued location, the highlight was immediately removed and after 180 ms, a highlight appeared at the next fixation in the sequence. (If the subject did not saccade to the cue after 3 s, the next cue was presented. Although this event was rare, a cue timeout guaranteed the experiment would not extend beyond the allotted time. A saccade to a cue might not be registered because the subject could not locate it or the eye tracker could not get an appropriate reading.) The highlighted training proceeded until all fixations in the sequence had been visited in the correct order. The trial ended when the last fixation in the sequence was visited. Each trial lasted roughly 30 sec. By following the cues, the subjects traced out their assigned expert’s scanpath.

Each cue had a Gaussian-distributed intensity and filled only the white background of the image and did not obstruct the black fingerprint ridges. The center of the cue is specified by the expert’s fixation, and is fully saturated red. The cue has standard deviation of 0.25° of visual angle (10 pixels) and flashed once per second, with an onset-to-offset interval of 300 ms, to facilitate its detection. Onsets and offsets are strong attractors of attention regardless of the task [40,41].

Following the training phase, subjects were presented with on-screen instructions informing them that they would be comparing fingerprints without assistance (as they had done during the pre-training phase). The post-training phase consisted of a recorded 10 s free gaze period identical to the pre-training period. The stimulus order in the post-training was independently randomized from the pre-training stimulus order. The entire experiment took about 45 minutes.

In each phase of the experiment, each subject’s gaze was recorded by the Tobii T60 XL at the rate of 60 Hz. Along with the eye coordinates at the time of sampling, the Tobii records a code for each eye indicating whether or not the eye has been detected. The data were cleaned by removing invalid gaze points. Although the Tobii is able to repair samples that have only one invalid eye, samples that have two invalid eyes must be thrown out. Any trials that have over 50% invalid gaze samples are thrown out since this suggests a systematic problem with the trial. While our rejection criterion is low, in practice, nearly all subjects in the following experiments had greater than 90% valid gaze samples. Gaze collection issues tended to concentrate with specific individuals, resulting in a small number of subjects being dropped from further analysis. Following the removal of invalid gaze points, the location of the gaze points is checked to make sure that all gaze points fall on the stimulus. Since the stimuli do not fill the entire screen of the eye tracker, it is possible for subjects to look off the stimulus. Any samples that are off the stimulus are removed and trials that have more than 50% of the gaze points off the stimulus are thrown out because this suggests a systematic inattention to the task.

Since the eye-tracker collects data at a uniform rate and does not directly determine fixations and saccades, it is necessary to run the gaze data through a fixation filter to extract fixation locations. The gaze filter used follows common parameters used in other research [35,42]. Specifically, a median filter with a window of three consecutive points is run over the data, which serves to reduce noise in the data. The magnitude of the velocity is then calculated at every point in the smoothed data and a velocity threshold is used to segment successive gaze points into fixations. The velocity threshold used here was 10° s^*-1*^. A minimum of 67 ms is established for a set of gaze points to be grouped as a fixation in order to eliminate extremely brief fixations. A threshold of 67 ms was used to mirror the analysis of Busey et al. [35]. Using alternative threshold values between 50–150 ms does not qualitatively change the results of later analysis. One subject was replaced due to difficulty collecting sufficient valid data. All subjects used for analysis had 32 valid trials.

### Results

The experiment involved four conditions in a 2-by-2 design: the time of recording (before vs. after training) crossed with whether the stimuli were used for training or not (training vs. transfer stimuli). We performed three different analyses on the cleaned data: (1) an empirically based analysis that measures the spatial proximity of subject fixations to expert fixations, (2) a model-based analysis that evaluates the degree of expertise via expert and novice image-specific models, and (3) a summary analysis of gaze statistics. We discuss each in turn.

#### Empirical analysis of spatial proximity to expert fixations

**Fig 6:**
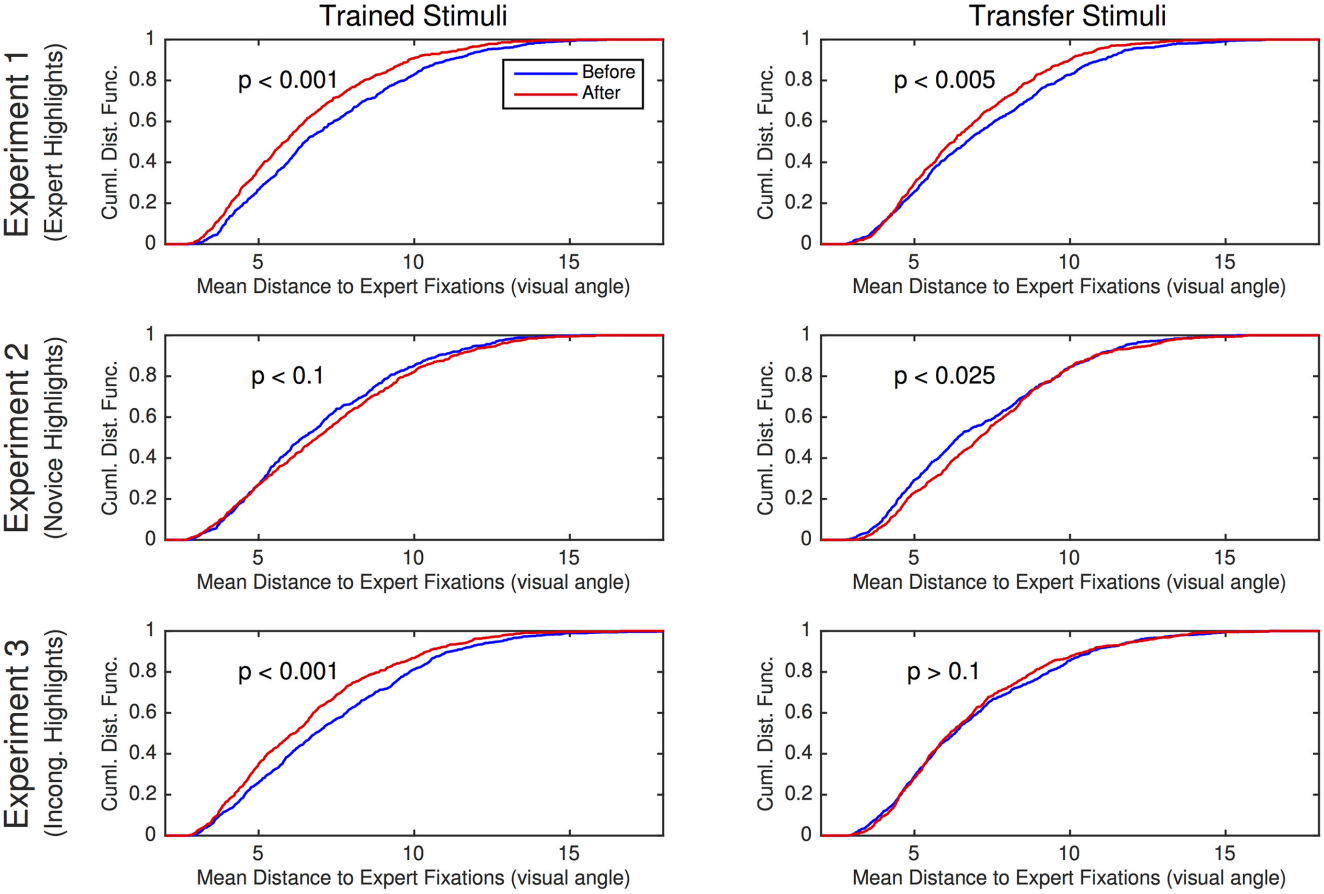
Summary of spatial proximity results from Experiments 1–3. Each experiment is shown in a row of the figure. The left and right columns present results for trained and transfer stimuli, respectively. Each graph shows the empirical cumulative distribution function before (blue) and after (red) the training phase. Each sample in the cumulative distribution function derives from the mean distance to all expert fixations (on the same image and impression) for a given subject fixation. Experiment 1–3 involved highlighted training based on expert fixations, novice fixations, and incongruent expert fixations, respectively.

For each fixation of each subject on a particular image, we determined the mean Euclidean distance to all expert fixations on the same fingerprint impression. This mean distance was used to form an empirical cumulative distribution function (CDF). By comparing the CDFs before and after training, we can assess the degree to which subjects’ fixations better approximated the experts’. The top row of Fig 6 shows the empirical CDF in the four conditions of Experiment 1. A two-sample Kolmogorov-Smirnov test (K-S test) allows us to test whether the probability distributions before and after training differ. If we treat each fixation as independent of the others, the K-S test produces highly significant effects for both the training stimuli (*D*(2025,  1913) = 0.114, *p* < .001) and the transfer stimuli (*D*(2049,  1825) = 0.083, *p* < .001). However, such a treatment doesn’t consider the dependence among fixations in a sequence and therefore inflates the apparent degrees of freedom in the data. Consequently, we propose an approach to estimating the reliability of differences in the distributions by excluding adjacent fixations, performing the K-S test with only every *k*-th fixation in a sequence—i.e., fixations 1, *k*+1, 2*k*+1. With a suitable *k*, the fixations will be independent. By intuition, we chose *k* = 3, which yields a reliable shift toward expertise for both training and transfer stimuli (*D*(706, 669) = 0.123, *p* < .001; *D*(718, 640) = 0.096, *p* < .005). Significance levels in Fig 6 are based on *k* = 3. Because of the arbitrariness in the choice of *k*, we performed a second, model-based analysis of the data.

#### Model-based analysis

**Fig 7:**
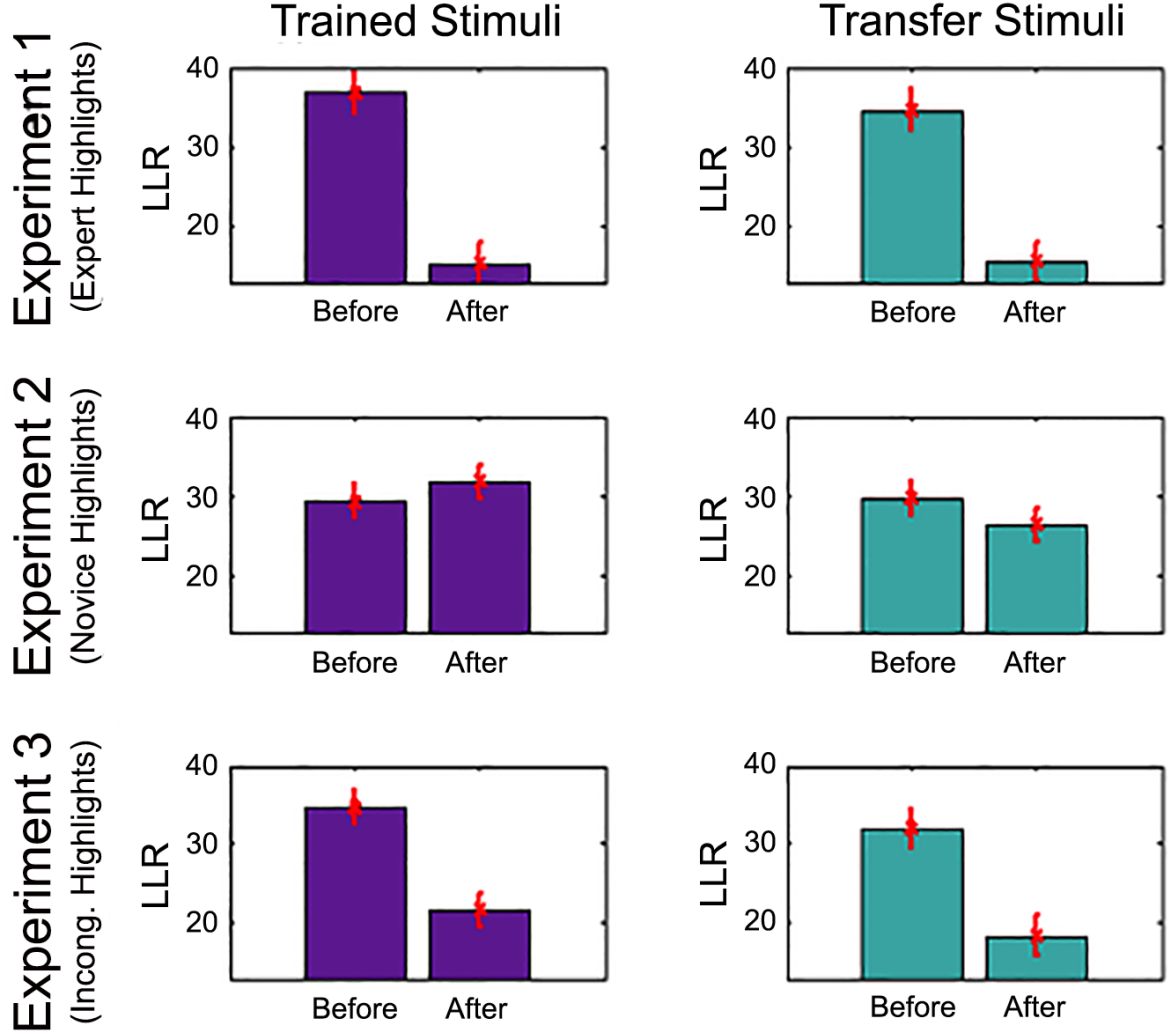
Summary of model-based analysis from Experiments 1–3. Each experiment is shown in a row of the figure, and the left column presents results for trained stimuli and the right column for transfer stimuli. The ordinate of each graph indicates the log likelihood ratio (LLR), a measure of *relative expertise*. An increase in the LLR from before to after training indicates acquisition of expertise. Experiment 1–3 involved highlighted training based on expert fixations, novice fixations, and incongruent expert fixations, respectively. The error bars are calculated to account for systematic tendencies of data derived from the same subject and variability between subjects [43].

The data were evaluated via the expert and novice image-specific models to obtain a log-likelihood ratio (the LLR, defined in Eq A5). The LLR provides a measure of *relative expertise*, allowing us to compare the nature of eye movements before and after training. The top row of Fig 7 shows relative expertise across subjects in the four conditions of Experiment 1. For both trained and transfer stimuli (left and right graphs, respectively), highlighted training shifts performance toward expertise. We conducted an analysis of variance (ANOVA) with subjects as the random variable and two within-subject factors: experiment phase (before vs. after training) and stimulus type (training, transfer). The ANOVA obtained a main effect of phase (*F*(1,  11) = 24.92, *p* < .001), no reliable effect of stimulus type (*F*(1,  11) = 0.05), and no phase-stimulus type interaction (*F*(1,  11) = 0.16). Thus, highlighted training was effective not only in altering fixation patterns on the specific images trained, but on novel images as well.

#### Summary gaze statistics

The data were evaluated to obtain low-level gaze statistics of various measures before and after training. This analysis included proportion of fixations on the left (vs. the right), proportion of saccades within impressions (vs. between impressions), mean fixation duration, mean saccade amplitude on the left and mean saccade amplitude on the right. Data were collapsed across training and transfer stimuli to reduce variance of the statistics. The top section of Table 1 shows the analysis of these variables for Experiment 1. There was a significant increase in the percentage of saccades that occurred within impressions; no other effect was significant.

**Table 1:** Low-level statistics of subject gaze behavior for Experiments 1, 2 and 3. For each experiment, an analysis was performed on the before- and after-training data for the following statistics: the percentage of fixations that occur on the left impression versus the right (*Left Fixations*), the percentage of saccades that occur within an impression versus across impressions (*Within Saccades*), the mean fixation duration (*Fixation Duration*), the amplitude of saccades that occur on the left and right impression (*Saccade Amplitude*). Experiment 1–3 involved highlighted training based on expert fixations, novice fixations, and incongruent expert fixations, respectively.

Characteristic	Before		After		Analysis			
	%		%		X^2^	*df*	N	*p*
Exp. 1								
Left Fixations	57.2		55.1		3.78	1	7812	.05
Within Saccades	68.3		71.3		7.62	1	7442	.006
	Mean	SD	Mean	SD	*t*	*df*		*p*
Fixation Duration (ms)	133.3	17.5	132.9	17.9	0.15	11		.880
Saccade Amplitude (°)								
Left	3.04	0.55	2.92	0.57	0.59	11		.570
Right	1.98	0.56	2.05	0.42	-0.28	11		.788
	%		%		X^2^	*df*	N	*p*
Exp. 2								
Left Fixations	52.6		52.2		0.15	1	6883	.703
Within Saccades	67.1		68.3		1.22	1	6509	.269
	Mean	SD	Mean	SD	*t*	*df*		*p*
Fixation Duration (ms)	140.7	30.3	145.7	28.9	-0.71	11		.492
Saccade Amplitude (°)								
Left	2.94	0.61	3.08	0.82	-0.72	11		.485
Right	2.69	0.94	2.51	0.61	0.71	11		.490
	%		%		X^2^	*df*	N	*p*
Exp. 3								
Left Fixations	51.9		52.8		0.70	1	8833	.70
Within Saccades	68.7		73.4		22.57	1	8461	< .001
	Mean	SD	Mean	SD	*t*	*df*		*p*
Fixation Duration (ms)	158.0	23.4	147.8	37.6	1.33	11		.211
Saccade Amplitude (°)								
Left	2.92	0.55	2.99	0.75	-0.28	11		.782
Right	2.41	0.38	2.39	0.72	0.14	11		.891

### Discussion

The results of Experiment 1 indicate that training via attentional highlighting leads to gaze patterns that are less like those of domain novices and more like those of domain experts. This shift toward expertise is observed both for stimuli that were used in training and for novel stimuli. Had highlighting benefited only the training stimuli, one might have explained the effect in terms of memorization of previous scanpaths [44]. However, both the empirical analysis and the model-based analysis indicate that generalization is quite robust, making it unlikely that subjects were simply memorizing the instructed fixation sequences.

The analysis of low-level gaze statistics provides some further support for the value of highlighting in facilitating the acquisition of expertise, but also points out some limitations. The increase in the percentage of saccades that occur within an impression is in agreement with the observations of Busey et al. [35] that experts make a higher percentage of saccades within impressions than do novices. It is possible that this reflects adoption of a more efficient examination strategy that seeks to compare an arrangement of features rather than compare features one at a time. In contrast, our analysis suggests that highlighting does not increase the percentage of fixations on the left or decrease the amplitude of saccades within an impression. Although Busey et al. [35] did find that experts fixated significantly more often on the left impression, the difference between experts and novices in their corpus was small (52.9% versus 51.4% respectively), and the reason for this difference with real-world experts is that examination strategies may focus on the latent print, which is conventionally placed on the left [35]. In our experiment, no latent print was used.

Thus far, we have focused on gaze statistics and haven’t reported measures of the primary discrimination task that subjects performed—comparing a pair of prints to determine if they came from the same individual. As we mentioned previously, 26 of the 28 images used in Experiment 1 contained matching prints. The predominance of matching prints ensures extended gaze sequences: with inked prints, the clarity of the images makes it easy to reject mismatching prints. Given a relatively small dataset and the imbalance of matches and mismatches, we did not expect any measure of discriminability or accuracy to provide reliable indications of learning, and thus did not collect same/different judgments.

One concern with Experiment 1 is that subjects might not be learning from the highlighting per se; rather, their improved performance might simply be due to mere exposure to the fingerprint stimuli and the resulting increased familiarity with fingerprints in general. To test this hypothesis, Experiment 2 was identical to Experiment 1, except that instead of highlighted training based on expert fixation sequences, the training procedure was based on novice fixation sequences. Each subject in Experiment 2 was matched with a novice in the training dataset, and the training novice provided the fixation sequences for highlighting. If highlighting has no effect, then Experiment 2 should produce the same results as Experiment 1. However, if subjects are learning from the highlighted sequence, the shift towards expertise should not be observed in Experiment 2.

## Experiment 2: Novice Highlighting

### Participants

Twelve new subjects were recruited from a paid subject pool at the University of Colorado, Boulder. All subjects had normal or corrected to normal vision and were given $10 in compensation for their time.

### Apparatus

The eye tracker setup and stimuli used were identical to Experiment 1.

### Procedure

Experiment 2 was identical to Experiment 1, except that highlighted training was based on fixation sequences from novices not experts. Subjects were not informed as to the source of the fixation sequences. The training and transfer stimuli used for the 12 subjects in Experiment 2 were matched to the training and transfer stimuli from the 12 subjects in Experiment 1, facilitating a comparison between the outcomes of the two experiments by removing one potential source of variation that differentiates the experiments. Each of the 12 subjects was paired in one-to-one correspondence with a random novice from the training dataset.

### Results

Data collected in Experiment 2 were cleaned and filtered for fixations in the same fashion as Experiment 1. One subject had 28 valid trials and one subject had 30 valid trials, but all remaining subjects had 32 valid trials. In the case where a trial is dropped, it is necessary to drop the corresponding trial in the pre-training or post-training period to balance the analysis.

#### Empirical analysis of spatial proximity to expert fixations

The empirical CDF of the mean distance to expert fixations before and after training on both training and transfer stimuli is presented in the second row of Fig 6. Evaluating the difference in distributions with a K-S test that uses every third fixation, as we did in Experiment 1, we find no reliable training effect on the training stimuli (*D*(599, 663) = 0.069, *p* > .05), and the effect on the test stimuli (*D*(564, 602) = 0.093, *p* < .025) is reliable, except in the opposite direction from Experiment 1: highlighted training using novice fixation sequences causes subjects to produce more novice-like fixations.

#### Model-based analysis

The relative expertise before and after training on both the training and transfer stimuli is presented in the second row of Fig 7. We conducted an ANOVA with subjects as the random variable and two within-subject factors: experiment phase (before vs. after training) and stimulus type (training, transfer). The ANOVA obtained no reliable effects of phase (F(1,  11) = 0.02) or stimulus type (F(1,  11) = 0.22). The phase-stimulus type interaction (F(1,  11) = 0.71) was also not significant.

An ANOVA was also conducted with the combined data from Experiments 1 and 2 to compare the effect of training with highlighting derived from expert versus novice fixation sequences. The ANOVA included highlighting source (expert versus novice) as a between-subjects factor, and phase of evaluation (before vs. after) and stimulus type (training vs. transfer) as within-subject factors. The ANOVA obtained a significant effect of phase (*F*(1,  22) = 18.66, *p* < .001) and an interaction of phase and source (*F*(1,  22) = 17.36, *p* < .001). Main effects and interactions involving stimulus type were not significant. Comparing the outcomes of Experiments 1 and 2 in Fig 7, one can see that the interaction is due to a training effect tending in opposite directions in the two experiments. Training on expert fixation sequences yields more expert-like behavior, and training on novice fixation sequences yields either no change or more novice-like behavior.

#### Summary gaze statistics

The analysis of low-level gaze statistics for Experiment 2 is presented in the second section of Table 1. None of the low-level gaze characteristics changed significantly as a result of highlighted training. In particular, training did not produce a significant increase in the number of saccades that occur within an impression (versus across impressions), in contrast to the effect of training in Experiment 1.

We note one caveat concerning the analysis that combines Experiments 1 and 2. The experiments were conducted consecutively in time, and thus subjects were not randomly assigned to be trained by expert vs. novice highlighting. However, the temporal lag between Experiments 1–3 was brief and a paid subject pool was used, and thus we are not concerned with systematic differences in the subject population across experiments.

### Discussion

The results of Experiment 2 indicate that training by highlighting novice fixation sequences does not yield more expert-like gaze sequences. Consequently, the training effect observed in Experiment 1 cannot be explained by mere exposure to and familiarity with the fingerprint stimuli. If the source of the training effect was exposure and familiarity and not the highlighting procedure, Experiments 1 and 2 would have produced identical results.

Although it’s conceivable that exposure might have some effect without any form of highlighting, Experiments 1 and 2 establish that subjects learn different fixation strategies as a result of expert-highlighted training versus novice-highlighted training. Nonetheless, we know little about what specifically is being learned. In our earlier characterization of expert fingerprint examiners, we discussed two spatial resolutions at which expertise is manifested. At the global scale, experts concentrate gaze on the center region of each fingerprint where the core and delta determine the gestalt flow of the ridges [34]. At the fine scale, experts are concerned with local configurations of visual features, such as the minutiae. Highlighting might train the attentional control system to be responsive to either or both of these constraints.

To discriminate among the possibilities, Experiment 3 was designed such that highlighted training provided guidance on only the coarse scale of fingerprint analysis and not on the fine scale, in contrast to highlighted training in Experiment 1, which provided guidance at both scales. If the training effect is reduced or eliminated in Experiment 3, we obtain evidence that highlighting facilitates the learning of task-relevant, spatially local visual features of the domain.

To eliminate fine-scale information in Experiment 3, highlighted training on a stimulus *X* was based on the fixation sequence of an expert for a different stimulus *Y*. This *incongruent* expert highlighting avoids cueing the locations of minutiae, which are fingerprint- and image-specific. However, it does cue coarse features of the fingerprints, for the following reason. Most fingerprints have a core and delta that occupy the center region of the fingerprint, and stimulus images are normalized on the screen; consequently, the locations of the cores and deltas for pair of stimuli, *X* and *Y*, are typically aligned.

## Experiment 3: Incongruent Expert Highlighting

### Participants

Twelve new subjects were recruited from a paid subject pool at the University of Colorado, Boulder. All subjects had normal or corrected to normal vision and were given $10 in compensation for their time.

### Apparatus and Materials

The eye tracker setup and stimuli used were identical to Experiment 1.

### Procedure

The design of Experiment 3 was identical to that of Experiment 1. Each subject was paired with an expert whose data provided the highlighting sequence. To match Experiment 1, Experiment 3 used the same subset of stimulus items for each expert, they were divided into training and transfer sets using the same split as Experiment 1, and the trial sequences of subjects in Experiment 3 matched those of subjects in Experiment 1. The key difference from Experiment 1 is that in Experiment 3, for each stimulus *X*, subjects were trained not on the expert fixation sequence corresponding to *X* but on the sequence corresponding to a different stimulus, *Y*. The re-pairing of expert fixations and stimuli was performed by permuting the set of stimuli such that each training stimulus was associated with the fixation sequence of a different training stimulus. Although one might ordinarily perform a permutation at random, we wanted to ensure that the reassigned fixation sequences were not by chance a good match to the incongruent stimulus. Consequently, the fixation sequences were permuted so as to minimize the likelihood of the incongruent fixation sequence under a model based on the congruent fixation sequence of all experts.

### Results

Data collected in Experiment 3 were cleaned and filtered for fixations in the same manner as Experiment 1. Only one subject had invalid trials (2 of 32 trials); therefore, no subject was rejected.

The bottom row of Fig 6 shows the empirical CDF in the four conditions of Experiment 3. The K-S test using every third fixation obtained a significant training effect for the training stimuli (*D*(875, 702) = 0.126, *p* < .001) but not for the transfer stimuli (*D*(843,  643) = 0.048, *p* > .10), suggesting that incongruent highlighted training is less effective than congruent training (Experiment 1).

Moving on to the model-based analysis, the relative expertise before and after training is presented in the third row of Fig 7. We conducted an ANOVA with subjects as the random variable and two within-subject factors: experiment phase (before vs. after training) and stimulus type (training, transfer). The ANOVA obtained a main effect of phase (*F*(1,  11) = 11.32, *p* = .006), no reliable effect of stimulus type (*F*(1,  11) = 1.02), and no phase-stimulus type interaction (*F*(1,  11) = 0.02).

An ANOVA was conducted with the combined data from Experiments 1 and 3 to compare the effect of highlighting training based on congruent versus incongruent expert fixation sequences. The ANOVA included congruency of highlighting (congruent in Experiment 1, incongruent in Experiment 3) as a between-subjects factor, and phase of evaluation (before vs. after) and stimulus type (training vs. transfer) as within-subject factors. The ANOVA obtained a significant effect of phase (*F*(1,  22) = 35.13, *p* < .001) and no significant interaction of phase and congruency (*F*(1,  22) = 1.57, *p* = .22). Main effects and interactions involving stimulus type were not significant. Although subjects were not randomly assigned to Experiment 1 versus Experiment 3—the three experiments were run in sequence—all subjects were drawn from a paid subject pool and participated early in the semester, which mitigates differences between the time each subject participated in the experiment and concerns regarding the influence of the end of the semester.

The third section of Table 1 shows the analysis of low-level gaze statistics for Experiment 3. There was a significant increase in the percentage of saccades that occurred within impressions. There was no significant difference for proportion of fixations on the left (vs. the right), mean fixation duration, mean saccade amplitude on the left and mean saccade amplitude on the right.

### Discussion

Experiment 3 was designed to examine the nature of the training effect. Via highlighted training with incongruent fixation sequences, Experiment 3 eliminated any systematic relationship between cued locations and local, fine-scale visual features of the fingerprint images (the minutiae) while preserving the relationship between cued locations and the global, coarse-scale visual features (e.g., the core and delta). Whereas Experiment 1 obtained strong evidence of generalization to novel stimuli, the evidence for generalization in Experiment 3 was weaker: the K-S test failed to show a shift in the fixation distribution for transfer stimuli with incongruent highlighting, but did show a shift with congruent highlighting. Given that highlights in Experiment 3 did not support the learning of fine-scale visual cues, our results hint that highlighting trains sensitivity to fine-scale information in stimuli. Although this conclusion is at best tentative, the comparison of Experiments 1 and 3 strongly suggests that subjects become sensitive to the coarse-scale information in fingerprint images as a result of highlighting training.

The comparison of Experiments 1 and 3 provides a hint that our design—a between-subject comparison with only 12 subjects per condition—may not have sufficient power to detect differences in the magnitude of the training effect between congruent (Experiment 1) and incongruent (Experiment 3) fixation sequences. And with only 64 training trials, the experiments might not have had sufficient duration to instill differences either. As a final caveat concerning the interpretation of our results, our dependent measure may not be adequately sensitive to subtle differences in expert behavior across stimuli. To understand why, we need to reconsider the modeling of novice and expert behavior. Free parameters of the models are optimized to discriminate novices and experts via the log-likelihood ratio (Eq A5). Although we’ve treated the models as if they can be used to assess expertise in general, the model fitting procedure is aimed at maximizing between-group discrimination, and it has no explicit requirement to distinguish between fine- and coarse-scale expertise. Indeed, the model bandwidth parameter forces a choice along the continuum of fine- to coarse-scale spatial representation, and the best fitting parameter value (a standard deviation of .5 degrees of visual angle) essentially blurs locations within the foveal region, forcing a scale of representation that might not be sufficiently sensitive to make the precise discriminations needed to evaluate fine-scale expertise. To test the conjecture that our model was simply not well-suited to discriminate coarse- and fine-scale expertise, we constructed a new model that was specifically designed to perform this discrimination. We define fine-scale expertise to be the pattern of fixations obtained from experts on a particular print. We define coarse-scale expertise to be the pattern of fixations obtained from experts combined across all prints. Since the images are fairly well-aligned, the coarse-scale expertise should reflect attention on the core and delta of a print. We obtain a log likelihood ratio that distinguishes between coarse and fine scale expertise, just as previously we obtained a log likelihood ratio that distinguishes between novices and experts. Using this dependent variable, one can ask whether Experiment 3 produced a weaker shift toward fine-scale expertise than did Experiment 1. Unfortunately, the outcome of this exploration was negative: it did not further distinguish the results of Experiments 1 and 3.

## Conclusions

Our work aims to understand the nature of attentional expertise by training subjects to fixate a series of locations via spatial cues and evaluating changes in gaze behavior as a consequence of training. Experiment 1 showed that highlighted training based on expert fixation sequences yields a shift toward expertise, not only for the trained stimuli but for novel stimuli as well. A modest number of training trials—8 trials on each of 8 images, each for about 30 s—was sufficient to yield appreciable behavioral changes. Experiment 2 provided a control to rule out the possibility that mere exposure or familiarity were responsible for the shift. Experiment 3 offered some insight into the nature of the shift: evidence was obtained for learning on a coarse spatial scale—the scale at which experts and novices are clearly discriminable—but we argued that limitations in our experimental design, dependent variable, and training procedure may explain why we failed to obtain strong evidence for learning about fine-grain stimulus structure.

A priori, it is not self-evident that highlighted training should modulate the deployment of attention: the procedure is not framed as a training task and any attentional adaptation is incidental. Subjects are instructed simply to follow the sequence of highlighted locations, and there is no explicit link between this task and the subsequent evaluation of behavior. Our results cannot be dismissed as reflecting pure priming whereby subjects memorize an association between images and fixation sequences because we observe generalization to novel images in Experiments 1–3. Even if subjects were attempting to memorize, scanpath variability is significant from one viewing to the next [45].

A limitation of our research is that we have not yet established a link between changes in gaze patterns and improved discrimination performance on the fingerprint-matching task. Nonetheless, behavioral changes may not be a particularly sensitive measure of acquired expertise. In a review of multiple studies of medical diagnosis, Reingold and Sheridan [11] discuss the general finding that cumulative cluster durations are longer for false-negative responses than true-negative responses. Even though final responses are negative (abnormality-free), gaze behavior yields a more sensitive measure of task processing. Differences between true-negative and false-negative response are robust enough that researchers have developed a visual feedback method to improve diagnostic accuracy [46].

Four modifications to the highlighted-training procedure may be beneficial. First, one might consider enhancing the discriminative visual features themselves, e.g., by increasing the contrast at locations containing minutiae. Second, one might explore a progressive training procedure in which subjects are first trained to locate and identify individual minutiae types and then training shifts to emphasize configural grouping of local features. Third, it may be useful to highlight not only the fixation location but also the spread or span of attention. In domains such as chess and medical imagery, experts do not necessarily foveate on the local features, but rather utilize parafoveal and peripheral vision to attend to local features in a larger spatial context [25]. Experts might fixate on the centroid of an arrangement of local features due to the learned expert unitization or chunking of co-occurring features [25]. The benefit of cueing novices to expert fixation locations presumes either that experts directly fixate minutiae or that a novice has a comparable visual span to perceive minutiae arrangements not directly fixated. Novices, with a smaller visual span, will lack the capacity to process minutiae that are not collocated at the fixation point [45]. To test this hypothesis, one could conduct further experiments that train visual span as well as fixation using local variability in expert fixations as a proxy for the appropriate visual span. Fourth, understanding the interplay of attentional and procedural expertise is beyond the scope of this article, but we anticipate that as the training technique is scaled up to more challenging tasks, such as matching latent prints, one might wish to interleave highlighted training with more traditional instruction on procedures and strategies, or perhaps even augment the highlighted locations with procedural information (e.g., change the color of a highlight to indicate regions of concern to the expert, as in [19]).

Our long-term objective is to design an approach that efficiently trains novices to perform complex visual tasks, leveraging the spatiotemporal dynamics of expert attention. The present work addresses two key challenges of this objective: modeling attentional expertise and assessing the effectiveness of highlighting in training subjects to fixate task-appropriate locations. However, a third key challenge remains: determining whether expert-like attention will aid in the classification or judgment task an individual must perform. Surely, attentional expertise will support some forms of procedural expertise. For example, in visual search, attentional training can guide novices toward locations whose contents are sufficiently interpretable and diagnostic that expert-like attention directly supports expert-like performance. Although visual search characterizes some key tasks requiring expertise (e.g., baggage screening), fingerprint analysis is more complex, involving temporally extended fixation sequences and information integration. Analyzing latent prints, which have missing and corrupted information, requires an additional degree of inference. In such tasks, the link between attentional expertise and procedural expertise is less direct. Nonetheless, as we discussed in the introduction to this article, past work provides encouraging indications that steering attention can guide higher-order cognitive processing. Expert-like attention should certainly support learning how to utilize critical visual information in a display, and therefore should facilitate the speed or ease with which expert performance is attained.

It remains for future research to explore the relative efficiency of highlighting versus more traditional training methods such as explicit instruction. However, highlighting is an attractive training tool because it enables attentional expertise to be shared with novices efficiently and effortlessly while circumventing complications regarding the articulation of visual expertise. Since the highlighting technique employed in this study has little dependency on the domain, it would be straightforward to explore training of expertise in other domains. Many domains could benefit from such training, including those involving visual search (e.g., baggage screening, satellite and medical imagery analysis), sequential decision-making (e.g., airplane piloting, air traffic control, driving), classification (e.g., detecting skin abnormalities), and highlighting might benefit not only typical individuals but also individuals with cognitive impairments (e.g., to train face and emotion recognition by autistic individuals).

### Using Models to Understand the Nature of Attentional Expertise

Although our primary focus was on evaluating the benefits of attentional highlighting, the models we constructed to perform this evaluation also offer insight into the nature of attentional expertise. As described in the S1 Appendix, we compared models that considered the duration of fixations to models that treated all fixations as equivalent. We had expected that the more critical locations would be fixated longer, and thus would be more predictive of expertise. However, we found that consideration of fixation duration provided no leverage in discriminating novice and expert behavior.

Fixation duration is only one means of incorporating time into models. The image-specific models we used to judge expertise predict the spatial distribution of attention by placing a Gaussian blob (kernel) of probability at the x-y location of each fixation. Such a model can be extended to the spatiotemporal domain via a 3D blob that extends in time as well as space. However, such a spatiotemporal model does no better than the pure spatial model in predicting expertise in the Busey et al. [35] data, even when the blob is allowed to spread broadly in time. This spatiotemporal model makes the strong assumption that individuals examine images in roughly a fixed temporal order. The model’s failure to perform better than the pure spatial model suggests that fixation order is relatively flexible.

Nonetheless, there are other means of incorporating sequential information into a model, such as considering the distribution of fixations with respect to the current fixation. In contrast to the spatiotemporal model that assumes locations are visited in a fixed order, this *relative-fixation* formulation claims merely that locations are visited based on their proximity to the current fixation. Proximity weighted gaze models have been constructed by incorporating an oculomotor bias favoring nearby fixations [47,48]. In order to model skill level, one might assume that the proximity bias is affected by expertise. In order to model the fingerprint-matching task, one might assume an additional bias in favor of saccades to roughly the corresponding location on the fingerprint not currently fixated. Our initial simulations strongly suggest that incorporating a proximity-weighted bias and a switch-to-other-print bias leads to better prediction of subsequent fixations. Further research will determine if including such sequential constraints leads to improved discrimination of expertise.

## Supporting Information

S1 Appendix Description of discriminative novice/expert model. (DOCX)
