# Correction: Using Highlighting to Train Attentional Expertise

**DOI:** 10.1371/journal.pone.0149368

**Published:** 2016-02-09

**Authors:** Brett Roads, Michael C. Mozer, Thomas A. Busey

The following information is missing from the Funding section: Publication of this article was funded by the University of Colorado Boulder Libraries Open Access Fund.
