# Supplementary material for: Using Highlighting to Train Attentional Expertise
Source: PLoS One. 2016 Jan 8;11(1):e0146266. doi: 10.1371/journal.pone.0146266 (PMC4706445; doi:10.1371/journal.pone.0146266)
Supplement: S1 Appendix — (DOCX) [file pone.0146266.s001.docx]

# S1 Appendix

## Discriminative Modeling of Novice and Expert Fixation Sequences

This appendix describes the construction of probabilistic models of novice and expert fixation sequences. A probabilistic model will assign a likelihood, $p\left( \boldsymbol{F} | s,\boldsymbol{I} \right),$ to a sequence of fixations, $\boldsymbol{F}$, given an image, $\boldsymbol{I}$, and a level of performance, $s\in\{novice, expert\}$. We describe and evaluate a variety of potential models, select the best model, and use the resulting novice and expert models to obtain a discriminative measure, the *degree of expertise* exhibited by an individual based on their fixation sequences. The degree of expertise is used in experimental studies to evaluate the effect of training.

We start by describing two broad classes of models. One class predicts the likelihood of any fixation sequence in *any* fingerprint image. We will call such a model an*image general model* (*IGM*). An IGM extracts visual features from the image and uses these features to determine the likely sequence of fixation locations. Using machine-learning tools, one could construct such a model from a set of example images and a set of example fixation sequences observed for those images. The resulting model could make predictions about fixation sequences for novel images. Although such a model is highly desirable, it demands a large dataset and further assumptions concerning feature extraction. As an alternative, in the present work, we use an *image specific model* (*ISM*) which ignores visual features but instead models the likely sequence of locations for a specific image.

In our modeling, we make the further assumption that the set of fixation locations in a sequence are chosen independently of one another, i.e.,

$p\left( \boldsymbol{F} | s,\boldsymbol{I} \right)=\prod_{i} p\left( \boldsymbol{f}_{i} | s,\boldsymbol{I} \right)$ (A1)


where *i* is an index over the fixation sequence and **f***_i_* denotes a single fixation. This assumption is certainly incorrect, but it makes modeling tractable and we’ve found in practice that even without sequential information, the models are sufficiently powerful to discriminate novices and experts. Perhaps this is due to the high variability in the order that fixations occur [1]. Under the independence assumption, a straightforward way to construct an ISM for a population, say experts, is to combine the set of fixations observed for the population on a specific image into a *kernel density estimate* (*KDE*). A KDE places a bump of probability mass at the location of every fixation. We assume that the bumps are Gaussians with a spherical covariance matrix, meaning that the bandwidth along the x-dimension is equal to the bandwidth along the y-dimension, and is specified by a single free parameter, *σ*. The KDE is normalized such that the total area under the mixture of Gaussian components integrates to one, yielding a probability density. Examples of an expert and a novice KDE with a bandwidth of approximately 0.5 degrees of visual angle are shown in Fig. A1. Given a location in the image, the KDE can be used to estimate the likelihood that an individual will fixate at that location.

The reader may be familiar with the related Gaussian mixture model (GMM), which is also a probability density formed from a mixture of Gaussian components. Typically, the number of GMM components is much smaller than the number of data points, and the mean and covariance of each component are fit to the data. Although the KDE might appear the be a more complex model, in that it has as many components as there are data points (fixations), it actually has a far smaller number of parameters to be fit, because the KDE component means are set directly from the data and the covariances are assumed to be identical for all components. We explored GMM fits to the data but found that they were not nearly as useful for our goal, which is to assess expertise of an unknown subject. We suspect that the problem with GMMs is that they lump together fixations on distinct minutiae and therefore are unable to discriminate fixation on the minutiae versus regions between minutiae.

Because the data set used to form the KDE is finite and many locations in the image have near zero likelihoods, we regularize the KDE model (i.e., make it more robust to the variability in the training sample) by mixing it with a uniform distribution. This mixture makes the generative claim that while a fixation is likely to fall around locations where others have looked, with a small probability, *c_s_*, viewers in population *s* pick an arbitrary location on the image. In total, the resulting ISM for population *s* has two free parameters: the bandwidth parameter *σ_s_* and the regularization coefficient *c_s_*. The ISM then specifies the likelihood of a fixation sequence ***F*** conditioned on population *s* based on Equation A1:

$p\left( \boldsymbol{F} | s,\boldsymbol{I} \right)\equiv\prod_{i} \left[ c_{s}\mathcal{U+}\left( 1-c_{s} \right)\sum_{k=1}^{K_{s}} \pi_{ks}\mathcal{N}\left( \boldsymbol{f}_{i};\mu_{ks},\sigma_{s} \right) \right]$ (A2)

where $\mathcal{U}$ is the uniform distribution and $\mathcal{N}$ is the normal (Gaussian). The KDE is specified by the number of mixture components (*K_s_*) in the Gaussian mixture, the relative weighting of the *k*th mixture component for image ***I*** (*π_ks_*), the spatial coordinates (*µ_ks_*) of the *k*th component, and the bandwidth parameter (*σ_s_*) in the spherical covariance matrix for population *s*. For the KDE model, *K_s_* is the number of fixations.

We considered two possibilities for the mixture weights. First, we supposed that all fixations are treated as equally important, in which case the relative weighting of each mixture component is given by *π_k_* = 1/*K_s_*. Second, we supposed that the importance of a fixation depends on the duration at which the eyes dwell on a location. To be precise, each fixation **f***_i_* is characterized by a location (*f_xi_*, *f_yi_*) and a duration *f_di_* such that the relative weighting of the *k*th fixation of *n* could be given by

$\pi_{k}=\frac{f_{dk}}{\sum_{i=1}^{n} f_{di}}$ (A3)


where *i* is an index over all fixations used to form the ISM.

A priori it is not clear which of the two fixation weighting schemes to utilize. Although dwell time has been shown to reflect ongoing perceptual and cognitive processing in domains such as reading [2], it is less clear to what extent fixation duration indexes moment-to-moment attentional processing at the point of fixation versus contributions from other types of processing such as saccade planning or parafoveal processing. However, dwell times have been shown to be diagnostic of false-negative versus true-negative responses for a variety of medical images such as mammography [3] and bone trauma [4], indicating that dwell times reflect at least some level of task-relevant attentional processing. Because it is difficult to adequately justify one weighting scheme over the other a priori, we consider both schemes in the model selection process.

## Measure of Expertise Using Image Specific Models

The purpose of the models we’ve described is to evaluate a fixation sequence from an unknown individual and assess the degree of expertise that the individual exhibits. The posterior probability of expertise in Equation 1 can be used to provide this assessment, but an equivalent formulation arises when the prior probabilities of novice and expert are both set to 0.5. In this case, the log likelihood ratio (LLR) conveys the same information as Equation 1:

$LLR\left( \boldsymbol{F} | \boldsymbol{I} \right)=\sum_{i=1}^{n} w_{i}\left[ \ln p\left( \boldsymbol{f}_{i} | s=novice,\boldsymbol{I} \right)-\ln p\left( \boldsymbol{f}_{i} | s=expert,\boldsymbol{I} \right) \right]$ (A4)


where the weights *w_i_* will be explained shortly. (The logarithm of the likelihood is used to compress the scale of the measure.) The LLR is 0 if the novice ISM and the expert ISM both score the sequence ***F*** as being equally likely; the LLR is positive if ***F*** is scored as being more novice-like and negative if ***F*** is scored as being more expert-like. The magnitude of the LLR reflects confidence in the classification, and can be used to observe incremental changes in expertise.

To this point, we have presented calculations for a single image, but the approach is readily extended to accommodate data from a subject over multiple images via:

$LLR=\frac{1}{S}\sum_{j} LLR\left( \boldsymbol{F}_{j} | \boldsymbol{I}_{j} \right)$ (A5)


where *S* is the number of images and *j* is an index over images and fixation sequences. Because fixation sequences will have different lengths and we wish to give equal emphasis to each trial regardless of the sequence length, *n_j_*, we set the weight *w_i_* = 1 ⁄ *n_j_* in Equation A4. (For duration-weighted modeling we scaled the influence of a single fixation by the duration of fixation relative to sum of the duration of fixations in the sequence.)

## Training and Validating an Image Specific Model

The log-likelihood ratio is a useful measure only if it can reliably discriminate between expert and novice gaze behavior. In this section, we describe how we train models and then validate their quality, in order to justify the use of these models in measuring changes in expertise resulting from experimental manipulations.

Because we are using ISMs, we require 2*N* models for *N* images―one for each of the two (novice and expert) populations. In principle, each model has 2 free parameters (*σ_s_*, *c_s_*) beyond the kernel centers, which are specified by a set of fixation points. Although models are image specific, the parameters reflect characteristics of an individual’s eye-movement control system, and thus should not vary across images. Consequently, the number of parameters is independent of the number of stimuli.

The remaining parameters can be constrained in three different ways. In a *4-parameter model*, the regularization coefficient (*c_s_*) and bandwidth parameter (*σ_s_*) are specific to the population. Allowing the regularization coefficient to depend on expertise follows the intuition that novices will be more likely to go ‘off task’ and make haphazard gaze choices; and allowing the bandwidth parameter to depend on expertise captures the notion that the degree of specificity in gaze choice may be lower for novices. In a *3-parameter model*, the regularization coefficient is modulated by expertise but the bandwidth parameter is shared between experts and novices. This model is based on the intuition that gaze accuracy depends on noise in the attentional and saccade generation system that is independent of expertise. Finally, in a *2-parameter model*, both regularization coefficient and bandwidth parameter are shared between experts and novices. This 2-parameter formulation supposes that a difference arises between novices and experts only in the kernels (the fixation locations). We consider these three different parameterizations, and for each, we consider two schemes for weighting fixations (equal or duration weighted), for a total of six distinct models.

Fitting a model means tuning parameters to maximize the likelihood of the training examples. Specifically, if a fixation sequence ***F****_j_* is that of a novice, then parameters should be found that maximize *LLR*(***F****_j_* | ***I****_j_*) (Equation A4), or if ***F****_j_* is that of an expert, then the parameters should maximize $-LLR(\boldsymbol{F}j | \boldsymbol{I}j)$. We therefore use a variant of Equation A5 as our objective function. Constrained nonlinear optimization is performed using the Nelder-Mead simplex algorithm [5] with the constraints that bandwidth parameters cannot be less than 0.5° of visual angle, which is the approximate lower bound error reported for the training data [6], and the regularization coefficient is bounded by the interval [0, 1].

To validate the models, we perform cross validation by splitting the training dataset into a hold-in set and a hold-out set. The hold-in set consists of the fixation data of eleven experts and eleven novices. The hold-out set consists of the remaining expert and novice fixation data and can be used to predict model performance on unseen data via the same LLR measure as used for training.  The training dataset can be split into a hold-in and hold-out set 144 different ways. For each split the hold-out data is evaluated in two ways. The first is a binary *ranking measure* that asks if the hold-out expert has a more expert-like ratio than the hold-out novice. We evaluate all 144 different hold-in/hold-out splits. The second test performs *individual classification*, labeling each hold-out subject as an expert or novice, creating 288 different test cases. The results of this hold-one-out cross validation procedure are shown in Table A1. According to the ranking measure, the 2- and 3-parameter formulations perform equally well regardless of the weighting scheme (equal weighting or weighting by fixation duration). However, the individual classification measure reveals that the 2-parameter formulation is more appropriate. Models with more free parameters perform worse, suggesting overfitting of the training data. Therefore the most parsimonious model is the 2-parameter formulation. Because the weighting scheme does not seem to affect performance for the 2-parameter model, we prefer the simpler model that gives equal weighting to all fixations. This model appears to do very well in classifying unknown individuals as novices or experts, with 97.92% accuracy in ranking two individuals as more expert like or more novice-like, and with a 92.01% accuracy of classifying an individual as novice or expert.

We trained the selected 2-parameter model on the entire dataset, yielding 10^-148^ and 0.500° for the regularization and bandwidth parameters. This new fully trained model is used to evaluate gaze data in the experiments that follow. We also leverage the LLR scores for held-out novices and experts in the 144 validation splits; the mean LLR scores are 24.7 and −9.6 for novices and experts, respectively, reflecting a clear differentiation. We use these mean LLR scores to scale the scores of subjects in our experiments, in order to determine where they lie on the continuum of novice to expert fixation behavior.


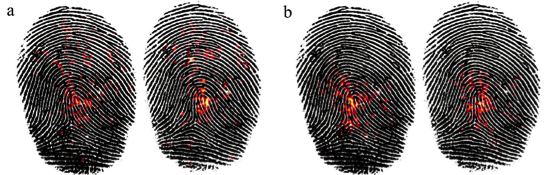


Fig. A1. **A kernel density estimate of novices (a) and experts (b) on the same stimulus.** Each KDE is constructed using fixations from twelve subjects of the respective population who viewed this stimulus for 10 s. Each fixation has a Gaussian bump centered on it with a bandwidth of approximately 0.5 degrees of visual angle. The color overlaid on the profile of the print corresponds to the likelihood a fixation would be generated at that location. From lowest probability to highest probability is black, dark red, red, orange, yellow and light yellow.

Table A1

Results of a hold-one-out test procedure

| Weighting Scheme | | Equal Weight | | | Weighting by Duration | | | |  |
| --- | --- | --- | --- | --- | --- | --- | --- | --- | --- |
| Parameter Formulation | | 2 | 3 | 4 | | 2 | 3 | 4 | |
| Ranking Measure (%) | | **97.92** | 97.92 | 96.53 | | **97.92** | 97.92 | 96.53 | |
| Classification Measure (%) | |  |  |  | |  |  |  | |
|  | Expert | 85.42 | 76.39 | 35.42 | | 85.42 | 81.25 | 36.11 | |
|  | Novice | 98.61 | 100 | 100 | | 98.61 | 100 | 100 | |
|  | Combined | **92.01** | 88.19 | 67.71 | | **92.01** | 90.62 | 68.06 | |

Note: Results of a hold-one-out test procedure using a log-likelihood ratio after optimizing on two, three, and four parameter implementations of two weighting schemes. The accuracy of the ranking measure is determined by the correct ranking of the hold-out expert and novice based on their log-likelihood ratio and is comprised of 144 test cases. Individual classification is carried out by independently classifying all hold-out subjects, resulting in 288 test cases.

# References

1. Mannan SK, Ruddock KH, Wooding DS. Fixation sequences made during visual examination of briefly presented 2D images. Spatial Vision. 1997; 11(2): p. 157-178.

2. Rayner K. Eye movements in reading and information processing: 20 years of research. Psychological Bulletin. 1998; 124(3): p. 372-422.

3. Krupinski EA. Visual scanning patterns of radiologists searching mammograms. Academic Radiology. 1996; 3(2): p. 137-144.

4. Krupinski EA, Lund PJ. Differences in time to interpretation for evaluation of bone radiographs with monitor and film viewing. Academic Radiology. 1997; 4(3): p. 177-182.

5. Lagarias JC, Reeds JA, Wright MH, Wright PE. Convergence properties of the Nelder--Mead simplex method in low dimensions. SIAM Journal on Optimization. 1998; 9(1): p. 112-147.

x

6. Busey TA, Yu C, Wyatte D, Vanderkolk J. Temporal Sequences Quantify the Contributions of Individual Fixations in Complex Perceptual Matching Tasks. Cognitive Science. 2013; 37(4): p. 731-756.


x
